# An Apriori Algorithm-Based Association Rule Analysis to Identify Herb Combinations for Treating Uremic Pruritus Using Chinese Herbal Bath Therapy

**DOI:** 10.1155/2020/8854772

**Published:** 2020-11-23

**Authors:** Ping-Hsun Lu, Jui-Lin Keng, Ko-Li Kuo, Yu-Fang Wang, Yu-Chih Tai, Chan-Yen Kuo

**Affiliations:** ^1^Department of Chinese Medicine, Taipei Tzu Chi Hospital, Buddhist Tzu Chi Medical Foundation, New Taipei, Taiwan; ^2^School of Post-Baccalaureate Chinese Medicine, Tzu Chi University, Hualien, Taiwan; ^3^Department of Applied Mathematics, University of Washington, Seattle, WA, USA; ^4^Division of Nephrology, Department of Internal Medicine, Taipei Tzu Chi Hospital, Buddhist Tzu Chi Medical Foundation, New Taipei, Taiwan; ^5^School of Medicine, Tzu Chi University, Hualien, Taiwan; ^6^Department of Research, Taipei Tzu Chi Hospital, Buddhist Tzu Chi Medical Foundation, New Taipei City, Taiwan

## Abstract

Uremic pruritus (UP) is prevalent among patients with end-stage renal disease (ESRD), which causes severe itching and affects their quality of life. Additionally, patients experience fatigue and depression, and an increased risk of mortality has also been reported. A meta-analysis of 17 randomized controlled trials (RCTs) has indicated that Chinese herbal bath therapy (CHBT) had adjuvant benefits in improving UP in ESRD patients, and previous studies have reported that herb combinations were more useful than treatment with a single herb. Association rule analysis has been used to evaluate potential correlations between herb combinations, and Apriori algorithms are one of the most powerful machine-learning algorithms available for identifying associations within databases. Therefore, we used the Apriori algorithm to analyze association rules of potential core herb combinations for use in CHBT for UP treatment using data from a meta-analysis of 17 RCTs that used CHBT for UP treatment. Data on 43 CHBT herbs were extracted from 17 RCTs included for analysis and we found 19 association rules. The results indicated that the following herb combinations {Chuanxiong, Baijili} ≥ {Dahuang} and {Dahuang, Baijili} ≥ {Chuanxiong} were most strongly associated, implying that these herb combinations represent potential CHBT treatments for UP.

## 1. Introduction

Uremic pruritus (UP) is a common but annoying symptom among patients with advanced chronic kidney disease (CKD) or on dialysis; it affects 24% of nondialysis CKD patients and 55% of dialysis patients [1, 2]. UP may be generalized or localized and typically worsens at night, thereby affecting sleep quality. The persistence of these symptoms leads to patients becoming fatigued, anxious, and depressed. Furthermore, the risk of mortality increases due to chronic fatigue syndrome [3, 4]. The pathogenesis of UP is not fully understood but systemic inflammation and opioid system imbalance are thought to be important contributors [3, 5, 6]. Additionally, effective treatment strategies for UP are lacking as its pathophysiology is not completely understood. Current therapies like antihistamines, gabapentin, nalfurafine, and phototherapy not only have a limited therapeutic effect but are also associated with adverse effects, such as dizziness, drowsiness, and somnolence [7, 8]. Therefore, alternative treatments for UP are required.

Complementary and alternative medicine is an attractive treatment option for UP because it is cheap and can have fewer side effects [9]. Chinese herbal bath therapy (CHBT) is an ancient treatment method that has been broadly used in China for thousands of years to ameliorate symptoms of certain diseases, including UP in uremic patients [7, 8]. The medicinal ingredients used in CHBT can improve blood circulation and have been shown to have analgesic and anti-inflammatory effects [10,11]. A recent meta-analysis has revealed CHBT to be effective as an adjunct therapy for improving UP among ESRD patients and the possible underlying mechanisms include improved blood circulation to promote metabolism, elimination of uremia-associated toxins [7,12], and anti-inflammatory effects [10,13]. It is widely recognized that both selection and combination of herbs used in CHBT are crucial for its success, and the principles used for selecting and combining such herbs follow ancient theories provided in the Rhymed Discourse for Topical Remedies and prescriptions of Chinese Materia Medica [14, 15]. Nevertheless, there is no clear consensus on the standard of CHBT herbs, or their combinations, for use in UP treatment.

Data mining methods have recently gained broad application in Chinese medicine, and many studies have used data mining to provide benchmarks for selecting and combining herbs for treating CKD [16], bone marrow suppression [17], and breast cancer recurrence and metastasis [18]. Even though the clinical practice of prescribing herbal medications is based on combinations of herbs, criteria for determining requisite combinations are lacking. Thus, association rule analysis could be used as an essential and practical approach for exploring the fundamental rules that would yield effective herb combinations. In recent years, machine-learning algorithms have been applied in many fields, including Chinese medicine. Apriori, an association rule learning algorithm, can determine the frequency of sets of items in databases [19] and also the importance of association rules based on certain metrics, such as support, confidence, and lift. Apriori offers thorough association analysis and can provide insights into trends within a database [19].

Hence, we aimed to explore potential core herb combinations for CHBT that can be used in the treatment of UP using an Apriori algorithm-based association rule analysis on datasets derived from a meta-analysis of 17 randomized controlled trials (RCTs) [8].

## 2. Materials and Methods

### 2.1. Data Sources and Selection Criteria

This analysis was based on the previously reported meta-analysis of 17 RCTs on CHBT treatment for UP [8]. We extracted data on herbs used for CHBT from these 17 RCTs collected from 2004 to 2017.

This meta-analysis included 970 UP patients with advanced CKD or those who were on dialysis; patients were graded according to the diagnostic criteria of the Chinese diagnostic standard or National Kidney Foundation (NKF) [8]. All the included studies were required to have precise outcome data on UP assessment parameters and to have conducted the herbal bath with only the patient's head remaining out of the water. The exclusion criteria were the use of other traditional Chinese medicine treatments by the patients or incomplete outcome data. The CHBT prescription uses an average of 11 Chinese herbs; the average duration of CHBT treatment was 4.7 weeks. Compared with the control group, CHBT has significantly improved visual analog scale (VAS) and symptom scores.

### 2.2. Risk of Bias Assessment

The methodological quality of the studies included for the meta-analysis was assessed using the Cochrane risk of bias (RoB) 2.0 tool [20]. This tool has five domains for assessing the risk of bias and the responses to questions in each domain are combined to provide an estimate of the overall quality of the RCT. This quality assessment procedure has been described in detail previously [8].

### 2.3. Data Analysis

We investigated the frequency of use of Chinese medicinal herbs. The software RStudio (version 1.2.5033, Integrated Development for R. RStudio, PBC, Boston, MA) was used to conduct the Apriori association rule learning analysis and to plot charts [21]. Data was processed into 17 columns with each column representing one CHBT formula, and data were fitted using the R package “arules.” Additionally, charts were produced and visualized by fitting data into the R package, “arulesViz.”

Association rule learning algorithms are the most popular method of identifying and analyzing trends or relations within transaction data [22]. Many studies have utilized association rule learning algorithms to detect hidden relations in medical fields [16–18]. Generally, an Apriori algorithm connects an antecedent set of items to a consequent set of items based on the notion that these two sets of items only occur together in the database, rather than due to a causal effect.

Support, confidence, expected confidence, and lift are four standard metrics used to measure associations between items in the Apriori algorithm. Support measures the proportion of CHBT formulas in which a certain herb appears while confidence measures the proportion of CHBT formulas with herb A, in which herb B also appears. Next, expected confidence measures the percentage of occurrences containing the consequent in a relation and represents the probability of the consequent if it is independent of the antecedent. Lastly, lift is the results obtained when confidence is divided by expected confidence and represents the likelihood of an increase in the consequent given a particular antecedent. In other words, lift quantifies the probability of herb B appearing when herb A is present in a CHBT formula while controlling for expected confidence.

We analyzed the 19 best association rules with the minimum values for support and confidence being 5% and 90%, respectively. The reason for choosing these criteria was that the highest value for support was only 0.13636364. Additionally, the values of confidence for overall association rules were above 0.5. Based on the minimum requirements of the metrics, we filtered out less significant association rules and found that 19 association rules remained in the dataset. Lastly, as the confidence values for all 19 association rules in this study were unity, the association rules were sorted in descending order based on values obtained for the metric “support”.

## 3. Results

### 3.1. Study Characteristics and Risk of Bias Assessment

The methodologic quality of the retrieved studies is summarized in Table 1 and a detailed RoB assessment is provided in Supplementary Figure 1 [8]. The overall quality of the 17 RCTs was variable, with only two of them determined to be “moderate.” Possible reasons include the fact that only one article described a specific grouping method, none of the retrieved studies described allocation concealment, and only one trial used patient blinding.

### 3.2. Distribution of the Herb

**Figure 1:**
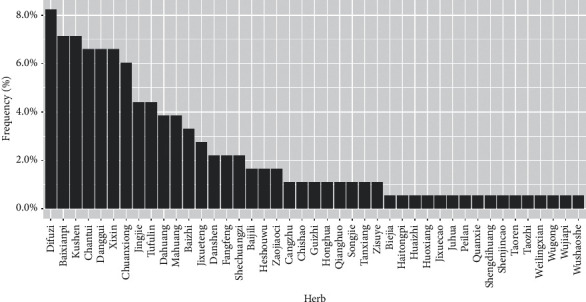
Frequency distribution of herbs used in the 17 RCTs included in the meta-analysis on CHBT treatment for UP.

We identified 43 herbs from the 17 RCTs included in the aforementioned meta-analysis.The frequency distribution of herbs is shown in Figure 1 and the Latin scientific names of the species are shown in Table 2. The 10 most-frequently selected herbs for use in CHBT for treating UP were Difuzi, Baixianpi, Kushen, Chantui, Danggui, Xixin, Chuanxiong, Jingjie, Tufulin, and Dahuang.

### 3.3. Apriori Algorithm-Based Association Rule Analysis for Item Sets of Herbs Combinations

**Figure 2:**
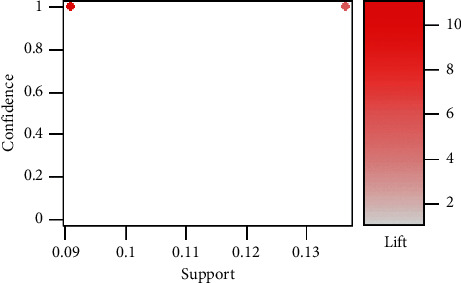
Scatter plot for the 19 association rules obtained in the 17 RCTs included in the meta-analysis on CHBT treatment for UP.

We analyzed 19 association rules based on data from 17 Chinese-medical-herb formulas. The association rules were converted to a scatter plot with support values on the *x*-axis and confidence values on the *y*-axis. The color of each association rule was determined by its lift value (Figure 2). We found that all association rules had a relatively high lift, indicating that the likelihood of antecedent herb and consequent herb being in an association rule together was multiple times that of the consequent herb when used alone. The confidence values for all 19 association rules was one, implying that each pair of the antecedent and the consequent herb appeared together whenever the antecedent herb was present in a formula in all 19 association rules. In contrast, only two values were obtained for support (0.13636364, 0.09090909); therefore, as seen in Figure 2, there was overlap among the 16 association rules on the left-hand side and among the three association rules on the right-hand side. These observations on support values revealed that the antecedent herb in each association rule did not frequently show in the 19 formulas. Thus, we deduced that each formula used for treating UP was implemented independently. All 19 association rules are listed in Table 3.

**Figure 3:**
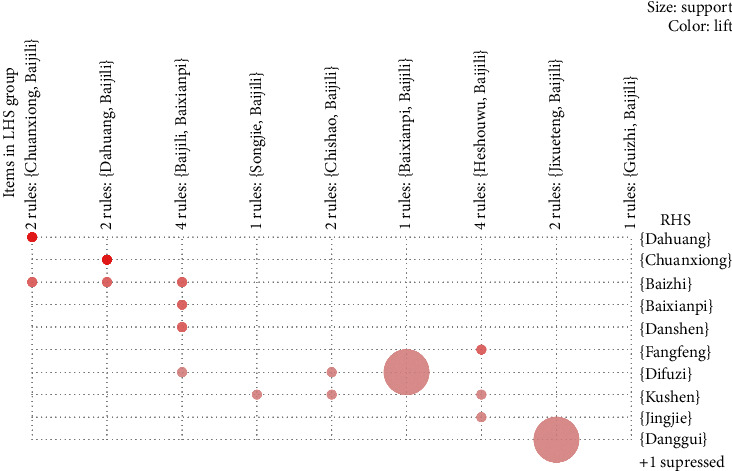
Grouping matrix for the 19 association rules obtained in the 17 RCTs included in the meta-analysis on CHBT treatment for UP.

A grouping matrix diagram, which displays the general distribution of clustered association rules, showed similar cluster-grouping of association rules (Figure 3). The horizontal ordinate represents 9 clusters, and the vertical ordinate stands for items produced by the 9 clusters (rules). The depth of color within a circle represents the degree of lift such that the darker the color, the higher the degree of lift. Circle size represents the degree of support such that the larger the circle, the higher the degree of support. The herb combinations {Chuanxiong, Baijili} ≥ {Dahuang}, {Dahuang, Baijili}≥{Chuanxiong} were interactively selected and they revealed the association rules connecting from antecedent (LHS) to consequent (RHS) item sets. The results of these interactively selected association rules consistently corresponded with association rules #4 {Chuanxiong} ≥ {Dahuang} and #5 {Dahuang} ≥ {Chuanxiong}, as seen in Table 3.

## 4. Discussion

Our results indicate that Chuanxiong, Baijili, and Dahuang, and Dahuang, Baijili, and Chuanxiong were the most-frequently used core herb combinations in CHBT for UP. Importantly, a meta-analysis of CHBT for UP revealed that the herb combination used in CHBT played a significant role in improving UP symptoms in patients with advanced CKD [8]. The usefulness of evidence-based strategies for selecting herbs for further treatment can be determined by their efficacy, and to the best of our knowledge, this is the first report on potential core herb combinations in CHBT for UP.

The current studies confirm that Chinese medicinal formulas containing herb combinations, when used orally or externally, are beneficial for patients with UP and that the possible underlying mechanisms include anti-inflammatory effects [10, 13], accelerating blood circulation to promote metabolism and eliminating uremia toxins [7, 12]. Chen et al. have reported a RCT that combined hemodialysis and an oral uremic clearance granule (UCG) containing 16 herbs, such as Chuanxiong, Dahuang, Kushen, Danshen, Heshouwu, and Sangbaipi, among others. They state that these herbs could not only significantly reduce itching when measured using a visual analog scale but also ameliorate renal function and reduce serum levels of phosphorus, calcium, intact parathyroid hormone (iPTH), and inflammatory biomarker, such as beta 2-microglobulin (*β*2-MG) [9]. Additionally, Kao et al. have revealed that a UCG-enema group showed a better effective rate for itch relief compared to the control group [40]. Wang et al. have demonstrated that Xiao Feng San (XFS), a traditional Chinese formula composed of 13 herbs, namely, Kushen, Chantui, Danggui, Fangfeng, and Jingjie, was better able to reduce pruritus and parathyroid hormone levels compared to control [41]. Bai et al. conducted an RCT using life paste, a Chinese herb-based cream that contains Dahuang, Danggui, Zicao, and Shechuangzi, and showed a better antipruritic effect for the herb combination than the lotion without the additives [42]. The Latin scientific names of the species mentioned above are shown in Table 2. The combination of *Astragalus* henryi Oliv. and Angelica sinensis (Oliv.) Diels (A&A) have been shown to improve renal blood flow in rats with acute ischemic renal injury [43] and to reduce capillary loss; the latter involved higher expression of the vascular endothelial growth factor (VEGF) [44].

Many studies have reported that herb pairs exhibit greater pharmacological efficacy than a single herb. Specifically, Ying et al. describe that the Alpinia oxyphylla-Schisandra chinensis herb pair (ASHP) has an anti-Alzheimer's disease effect via ameliorating abnormal changes in cognitive behavior, and that ASHP can improve the absorption of and slow down the elimination of bioactive ingredients, compared to either Schisandra chinensis or Alpinia oxyphylla alone, in a rat model of Alzheimer's disease [45]. Luo et al. have reported that Sprague-Dawley rats orally administered the Ephedra sinica Stapf-Cinnamomum cassia (L.) J. Presl herb pair could improve the pharmacological effects of Ephedra sinica Stapf and reduce its toxicity (apparent when used alone) by reducing tissue accumulation of ephedrine alkaloids [46]. Hence, herb pairs appear to show greater pharmacological efficacy than a single herb.

Research using modern pharmacological methods has demonstrated that the core herb combinations listed here could improve UP through their anti-inflammatory, antioxidant, and antibacterial properties and by accelerating blood circulation to promote metabolism and eliminating uremia toxins [8, 47–51] (Table 4).

## 5. Conclusions

Chinese medicinal formulas that use combinations of many herbs represent potential therapeutic options for some conditions; however, data on their reliability and mechanism of action are lacking. Our study shows the combinations of herbs used in Chinese medicine formulas, such as Chuanxiong, Baijili, and Dahuang, and Dahuang, Baijili, and Chuanxiong, may be potentially useful in the treatment of UP. Further experiments are needed to confirm these observations and to explore their mechanism(s) of action.

### 5.1. Limitations

Despite our results on potential core herb combinations for the treatment of UP, this study has a few limitations. First, the overall risk of bias was high in most RCTs included in the meta-analysis and none of the studies mentioned double-blinding. Thus, high RoB should be considered when interpreting these results. Next, most of the included RCTs did not have careful follow-up protocols, and the treatment duration only lasts two weeks in some RCTs. Hence, future studies should focus on establishing the long-term efficacy and safety of these core prescriptions for UP. Finally, as the mechanisms of action of these herb combinations are unclear, further basic and clinical studies are also needed.

## Figures and Tables

**Table 1:** Summary of 17 RCTs included in the meta-analysis on CHBT treatment for UP.

Study (year)	Study design	Inclusion criteria	Herbs	Overall bias
Du, 2009 [23]	RCT	HD	Tufulin, chuanxiong, baizhi, baixianpi, difuzi, kushen, jingjie, xixin, danggui, chantui	High

Du et al., 2004 [24]	RCT	HD	Tufulin, chuanxiong, baixianpi, difuzi, heshouwu, kushen, jingjie, xixin, danggui	High

Gao and Ye, 2012 [25]	RCT	HD	Tufulin, dahuang, baixianpi, difuzi, heshouwu, fangfeng, taoren, mahuang, danshen, jixueteng	High

Guo et al., 2009 [26]	RCT	HD	Dahuang, chuanxiong, danshen, baixianpi, difuzi, honghua, kushen, xixin, mahuang, danggui	High

Jia et al., 2012 [27]	RCT	HD	Dahuang, chuanxiong, baizhi, baixianpi, difuzi, zaojiaoci, kushen, xixin, danggui, chantui, jixueteng	High

Jia, Li and Fei, 2012 [28]	RCT	HD	Dahuang, chuanxiong, baizhi, baixianpi, difuzi, zaojiaoci, kushen, xixin, danggui, chantui, jixueteng	High

Lan, 2017 [29]	RCT	HD	Dahuang, chuanxiong, danshen, baixianpi, difuzi, honghua, kushen, xixin, mahuang, danggui	High

Lin, 2014 [30]	RCT	HD	Dahuang, chuanxiong, baizhi, baixianpi, difuzi, zaojiaoci, kushen, xixin, danggui, chantui, jixueteng	High

Shen, 2014 [31]	RCT	HD	Tufulin, danshen, baixianpi, quanxie, difuzi, heshouwu, kushen, xixin, shechuangzi, mahuang, danggui, wugong, chantui, biejia	High

Wang et al., 2013 [32]	RCT	HD	Dahuang, chuanxiong, baixianpi, difuzi, chishao, kushen, xixin, shechuangzi, juhua, danggui, chantui	High

Wen, 2007 [33]	RCT	HD	Tufulin, chuanxiong, baijili, difuzi, wushaoshe, jingjie, xixin, danggui, jixueteng	High

Yao, 2015 [34]	RCT	HD	Tufulin, baixianpi, difuzi, kushen, jingjie, chantui	High

Yu et al., 2017 [35]	RCT	HD	Tufulin, chuanxiong, baixianpi, difuzi, kushen, jingjie, zisuye, danggui, jixuecao, chantui	High

Zhang et al., 2014 [36]	RCT	HD	Tufulin, difuzi, baixianpi, kushen, chantui, jingjie	High

Zhang et al., 2012 [37]	RCT	HD	Baizhi, baijili, fangfeng, songjie, qianghuo, guizhi, jingjie, xixin, shechuangzi, mahuang, cangzhu, tanxiang, chantui	High

Zhao, 2011 [38]	RCT	HD	Wujiapi, baizhi, baijili, shenjincao, fangfeng, peilan, songjie, qianghuo, weilingxian, guizhi, taozhi, haitongpi, jingjie, xixin, shechuangzi, mahuang, zisuye, huaizhi, cangzhu, tanxiang, chantui, huoxiang	Some

Zheng, 2016 [39]	RCT	HD	Chuanxiong, shengdihuang, difuzi, chishao, fangfeng, kushen, mahuang, danggui, chantui	High

RCT = randomized controlled trial; CHBT = Chinese herbal bath therapy; UP = uremic pruritus; HD = hemodialysis.

**Table 2:** The Chinese herbs mentioned in the article.

Chinese name	English name	Latin name	Frequency of usage
The 43 Chinese herbs used in 17 prescriptions of CHBT			
Difuzi	Fructus kochiae	*Bassia scoparia* (L.) A.J.Scott	15
Baixianpi	Densefruit pittany root-bark	*Dictamnus albus* L.	13
Kushen	Lightyellow sophora	*Sophora flavescens* aiton	13
Danggui	Chinese angelica	*Angelica sinensis* (oliv.) diels	12
Xixin	Manchurian wildginger	*Asarum sieboldii* miq.	12
Chantui	Cicada slough	*Cryptotympana atrata* fabr.	12
Chuanxiong	Szechuan lovage rhizome	*Ligusticum striatum* DC.	11
Jingjie	Fineleaf schizonepeta herb	*Nepeta tenuifolia* benth	8
Tufulin	Rhizoma smilacis glabrae	*Smilax glabra* roxb	8
Dahuang	Rhubarb root and rhizome	*Rheum officinale* baill.	7
Mahuang	Ephedra equisetina	*Ephedra sinica* stapf	7
Baizhi	Dahurian angelica	*Bletilla striata* (thunb.) Rchb.f.	6
Jixueteng	Suberect spatholobus stem	*Millettia dielsiana* harms	5
Danshen	Salvia miltiorrhiza *f* alba	*Salvia miltiorrhiza* bunge	4
Shechuangzi	Common cnidium	*Cnidium monnieri* (L.) cusson	4
Fangfeng	Divaricate saposhnikovia	*Saposhnikovia divaricata* (turcz.) schischk.	4
Heshouwu	Tuber fleeceflower	*Reynoutria multiflora (Thunb.)* moldenke	3
Zaojiaoci	Spine of Chinese honeylocust	*Gleditsia sinensis* lam.	3
Baijili	Tribulus fruit	*Tribulus terrestris L*.	3
Cangzhu	Atractylodes japonica	*Atractylodes lancea* (thunb.) DC.	2
Guizhi	Cassiabarktree twig	*Cinnamomum cassia* (L.) J.Presl	2
Honghua	Safflower	*Carthamus tinctorius* L.	2
Zisuye	Perilla frutescens var acuta	*Perilla frutescens* (L.) britton	2
Songjie	Knotty pine wood	*Pinus tabuliformis* carrière	2
Chishao	Red peony root	*Paeonia lactiflora* pall.	2
Qianghuo	Notopterygium forbesii	*Notopterygium incisum* K.C.Ting ex H.T.Chang	2
Tanxiang	Sandalwood	*Santalum album* L.	2
Haitongpi	Oriental Variegated coralbean bark	*Erythrina variegata* L.	1
Huaizhi	Twig of Japanese pagodatree	*Styphnolobium japonicum* (L.) schott	1
Wugong	Centipede	*Scolopendra Subspinipes Mutilans*	1
Wushaoshe	Chinese rat snake	*Zaocys dhumnades* cantor	1
Biejia	Turtle shell	*Trionyx sinensis* wiegmann	1
Huoxiang	Wrinkled gianthyssop	*Pogostemon cablin* (blanco) benth.	1
Peilan	Eupatorium formosanum	*Eupatorium fortunei* turcz.	1
Taoren	Peach kernel	*Prunus persica* (L.) batsch	1
Juhua	Florists chrysanthemum flower	*Chrysanthemum morifolium* ramat.	1
Wujiapi	Acanthopanax senticosus	*Eleutherococcus nodiflorus* (dunn) S.Y.Hu	1
Shengdihuang	Fresh rehmannia root	*Rehmannia glutinosa* (gaertn.) DC.	1
Weilingxian	Chinese clematis	*Clematis chinensis* osbeck	1
Quanxie	Scorpion	*Buthus martensii* karsch.	1
Jixuecao	Asiatic pennywort	*Centella asiatica* (L.) urb.	1
Taozhi	Prunus davidiana	*Prunus persica* (L.) batsch	1
Shenjincao	Common clubmoss herb	*Lycopodium clavatum* L.	1

The other Chinese herbs mentioned in the article			
Sangbaipi	Folium mori albae	*Morus alba* L.	-
Zicao	Radix arnebiae	*Lithospermum erythrorhizon* siebold and zucc.	-
Huangqi	Radix astragali	*Astragalus henryi* oliv.	-
Yizhiren	Sharpleaf galangal	*Alpinia oxyphylla* miq.	-
Wuweizi	Schisandra sphenanthera	*Schisandra chinensis* (turcz.) baill.	-

CHBT = Chinese herbal bath therapy.

**Table 3:** Apriori algorithm-based association rules for herbs used in CHBT.

No	Association rules	Support	Confidence	Expected confidence	Lift
1	{Jixueteng} ≥ {danggui}	0.13636364	1.000000	0.181818182	5.500000
2	{Jixueteng} ≥ {chantui}	0.13636364	1.000000	0.318181833	3.142857
3	{Baixianpi} ≥ {difuzi}	0.13636364	1.000000	0.181818182	5.500000
4	{Chuanxiong} ≥ {dahuang}	0.09090909	1.000000	0.090909091	11.000000
5	{Dahuang} ≥ {chuanxiong}	0.09090909	1.000000	0.090909091	11.000000
6	{Chuanxiong} ≥ {baizhi}	0.09090909	1.000000	0.136363643	7.333333
7	{Dahuang} ≥ {baizhi}	0.09090909	1.000000	0.136363643	7.333333
8	{Baijili} ≥ {baizhi}	0.09090909	1.000000	0.136363643	7.333333
9	{Baijili} ≥ {baixianpi}	0.09090909	1.000000	0.136363643	7.333333
10	{Baijili} ≥ {danshen}	0.09090909	1.000000	0.136363643	7.333333
11	{Baijili} ≥ {difuzi}	0.09090909	1.000000	0.181818182	5.500000
12	{Guizhi} ≥ {chantui}	0.09090909	1.000000	0.318181833	3.142857
13	{Songjie} ≥ {kushen}	0.09090909	1.000000	0.181818182	5.500000
14	{Chishao} ≥ {difuzi}	0.09090909	1.000000	0.181818182	5.500000
15	{Chishao} ≥ {kushen}	0.09090909	1.000000	0.181818182	5.500000
16	{Heshouwu} ≥ {fangfeng}	0.09090909	1.000000	0.136363643	7.333333
17	{Heshouwu} ≥ {jingjie}	0.09090909	1.000000	0.181818182	5.500000
18	{Heshouwu} ≥ {kushen}	0.09090909	1.000000	0.181818182	5.500000
19	{Heshouwu} ≥ {chantui}	0.09090909	1.000000	0.318181833	3.142857

CHBT = Chinese herbal bath therapy.

**Table 4:** Potential efficacy of the core Chinese medicinal herbs of CHBT used for the treatment of uremic pruritus.

Chinese name	English/Latin name	Function	Active ingredients	Efficacy
Baijili [48, 50]	Tribulus fruit/Tribulus terrestris *L*.	Pruritus relief	Saponins, flavonoids, glycosides	Antihelmintic, antibacterial, and antiinflammatory activity

Chuanxiong [47, 51]	Szechuan lovage rhizome/Ligusticum striatum DC.	Activating blood circulation	Ferulic acid, senkyunolide, butylidenephthalide	Antithrombotic, antihypertensive, and vasodilator activity

Dahuang [49]	Rhubarb root and rhizome/Rheum officinale baill.	Promoting detoxification	Anthraquinones, Stilbenes	Antibacterial, anti-inflammatory, and free-radical scavenging activity

CHBT = Chinese herbal bath therapy.

## Data Availability

The data used to support the findings of this study are included within the article.

## Supplementary Materials

Supplementary Materials Supplementary Figure 1: risk of bias assessment of the retrieved RCTs.
