# Supplementary figures and images for: An Apriori Algorithm-Based Association Rule Analysis to Identify Herb Combinations for Treating Uremic Pruritus Using Chinese Herbal Bath Therapy
Source: Evid Based Complement Alternat Med. 2020 Nov 23;2020:8854772. doi: 10.1155/2020/8854772 (PMC7704140; doi:10.1155/2020/8854772)

**Supplementary Figure 1.** Risk of bias assessment of the retrieved RCTs.
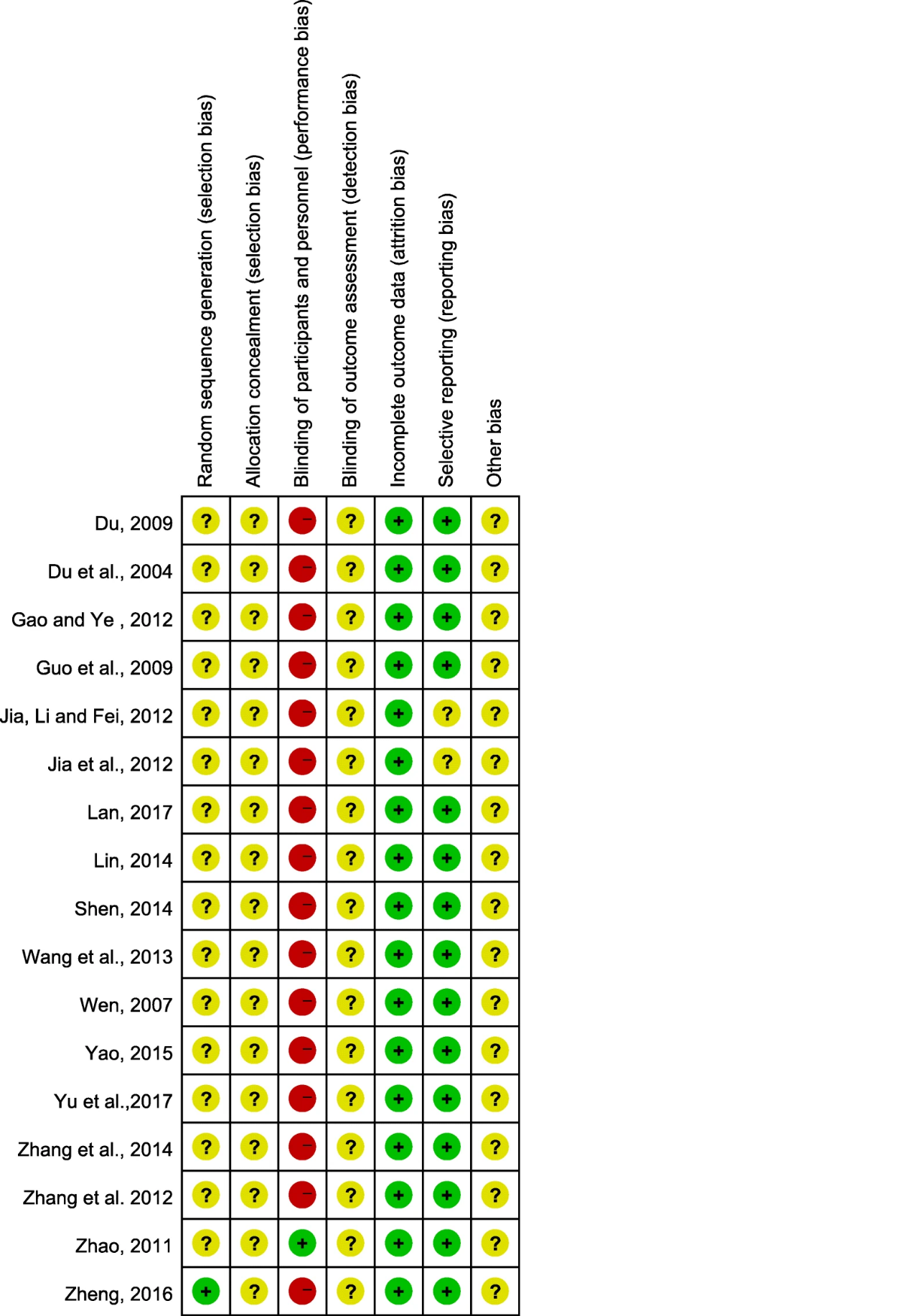

Supplement: Supplementary Materials — Supplementary Figure 1: risk of bias assessment of the retrieved RCTs. [file 8854772.f1.docx]
